# A Case of Ocular Sarcoidosis Post-COVID-19 Vaccination

**DOI:** 10.7759/cureus.49303

**Published:** 2023-11-23

**Authors:** Shreya Gandhi, Radhika Paranjpe, Ozukhil Radhakrishnan, Khushboo Goyal, Kalpita B Goli

**Affiliations:** 1 Ophthalmology, Dr. D. Y. Patil Medical College, Hospital & Research Centre, Pune, IND

**Keywords:** corticosteroids, multiorgan sarcoidosis, erythema nodosum, covid-19 vaccine, uveitis, autoimmune, granulomatous

## Abstract

A 35-year-old gentleman came to the ophthalmology outpatient department with complaints of bilateral ocular pain, redness and photophobia since three weeks with similar prior history. The patient was a diagnosed case of systemic sarcoidosis since two years with pulmonary, dermatological and neurological involvement for which he was already on treatment which included oral immunosuppressants, steroids, anticonvulsants and multivitamins. On examination, the best corrected visual acuity was 6/18 in the right eye and 6/12 in the left eye. On slit lamp and fundus examination, the patient showed signs of anterior and posterior uveitis in both eyes, the right eye more than the left eye. Treatment was initiated with topical corticosteroids and beta blockers and the patient improved following medical management.

## Introduction

Sarcoidosis is a chronic inflammatory disease affecting multiple body systems, characterised by the presence of non-caseating granulomas. Typically, its cause is unknown (idiopathic), but it has been associated with various factors such as bacterial, viral, fungal, and environmental agents. Additionally, certain agents like the coronavirus disease 2019 (COVID-19) vaccine, hepatitis C, and *Propionibacterium* have been implicated in its development [1].

Vaccine-induced uveitis has a complex pathogenesis, involving several mechanisms. These mechanisms include a close similarity between vaccine peptides and peptides in the uvea, inflammatory damage caused by aluminium salts, direct viral load (in live and attenuated vaccines), and type IV hypersensitivity with immune complex deposition. This condition usually affects both eyes, but in about 10% of cases, it may manifest unilaterally [2-4].

Ocular manifestations of sarcoidosis encompass a wide range of symptoms, including eyelid granulomas, lacrimal gland involvement, lesions of orbit and sclera, uveitis, vitritis, papilledema, papillitis, cataract, glaucoma, and others. The primary cause of visual impairment is typically maculopathy, with cystoid macular oedema being the most common underlying factor. Extraocular manifestations can include Lofgren syndrome, pulmonary fibrosis, meningitis, lymphadenopathy, and, rarely, cardiac arrhythmias [1,5].

Herein, we present an unusual case of ocular sarcoidosis that exhibited pulmonary, dermatological, and neurological manifestations. Corticosteroids served as the primary treatment approach.

## Case presentation

A 35-year-old male, an information technology specialist by profession, presented to the ophthalmology outpatient department with complaints of pain, redness, photophobia and ocular discomfort in right eye more than left eye since three weeks. He presented with a similar episode in the past two years back when he was treated with topical corticosteroids, beta blockers and mydriatics which provided symptomatic relief. The patient is a known case of neurosarcoidosis since two years with a history of having complaints of headache and generalised weakness for which treatment was started with oral medications; prednisolone 50 mg and vitamin B1 and B12 1 mg once a day, oxcarbazepine 450 mg and azathioprine 50 mg twice a day. The patient also gave a history of receiving Covishield COVID-19 vaccination two years back. He was diagnosed with erythema nodosum two years back and presented with hyperpigmented, erythematous plaques and nodules with discolouration on the right lower limb for which he was treated. He had complaints of transient blurring of vision one year back following which he was examined and fundus examination showed bilateral disc swelling. The patient had an intermittent cough for two months for which antitussives were prescribed and was gradually relieved.

**Figure 1:**
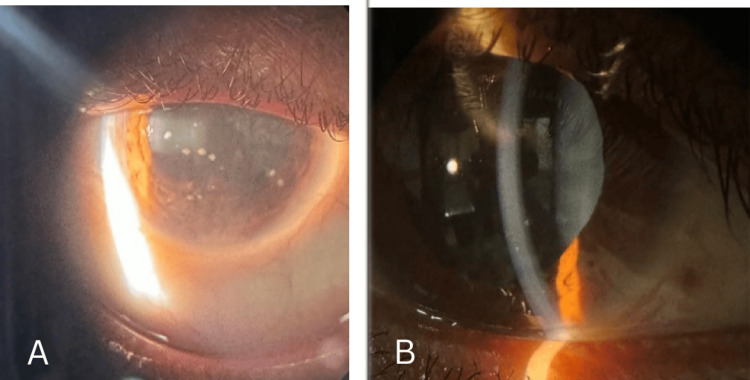
The image (A - right eye, B - left eye) shows a slit lamp photograph with the right eye showing circumcorneal congestion, epithelial oedema and mild stromal haze in the cornea, and mutton fat granulomatous keratic precipitates over the corneal endothelium in both eyes. Anterior chamber showed cells 2+ and posterior synechiae were noted inferiorly (at presentation) in the right eye and 1+ cells in the left eye (at presentation)

On ocular examination, the best corrected visual acuity was 6/18 and 6/12 in the right and left eye respectively. On slit lamp examination both eyes showed circumcorneal congestion, epithelial oedema and mild stromal haze in the cornea with mutton fat granulomatous deposits known as keratic precipitates (KPs) over the endothelium in the right eye more than left eye predominantly in the inferior quadrant. The anterior chamber showed cells 2+ in the right eye and 1+ in the left eye with no signs of flare and the anterior chamber depth was normal. No evidence of iris nodules was observed in either of the eyes. Posterior synechiae were seen in the right eye inferiorly and nasally and the pupil was central, irregular, reacting to light. The pupil was normal in the left eye with no synechiae formation. Lens showed early cataractous changes in both eyes (Figure 1).

**Figure 2:**
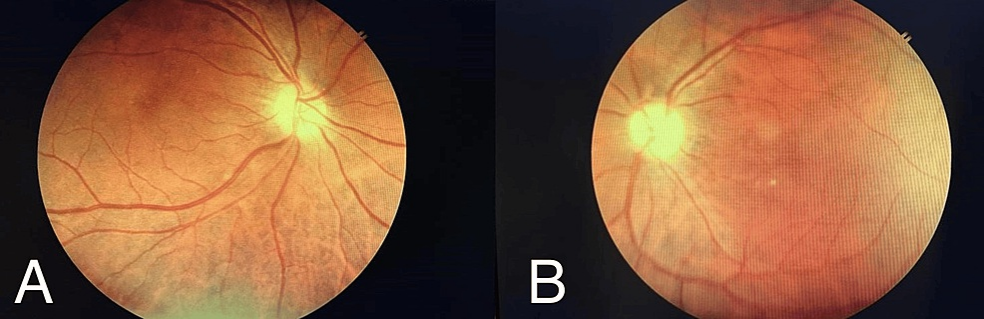
A shows a fundus photograph of the right eye showing periphlebitis along the superotemporal vascular arcade and multifocal choroiditis at the posterior pole with grade 1 vitritis. B shows perivenous sheathing along the superotemporal and inferotemporal vascular arcades with multifocal choroiditis at the posterior pole and mid periphery with grade 2 vitritis in the left eye on fundus photography

Intraocular pressure was measured using an applanation tonometer which was 28 mmHg in the right eye and 24 mmHg in the left eye. Fundus examination was performed with a 90D lens, direct and indirect ophthalmoscope which showed periphlebitis and multifocal choroiditis at the posterior pole in both the eyes with grade 1 and grade 2 vitritis in the right and left eye respectively (Figure 2).

**Figure 3:**
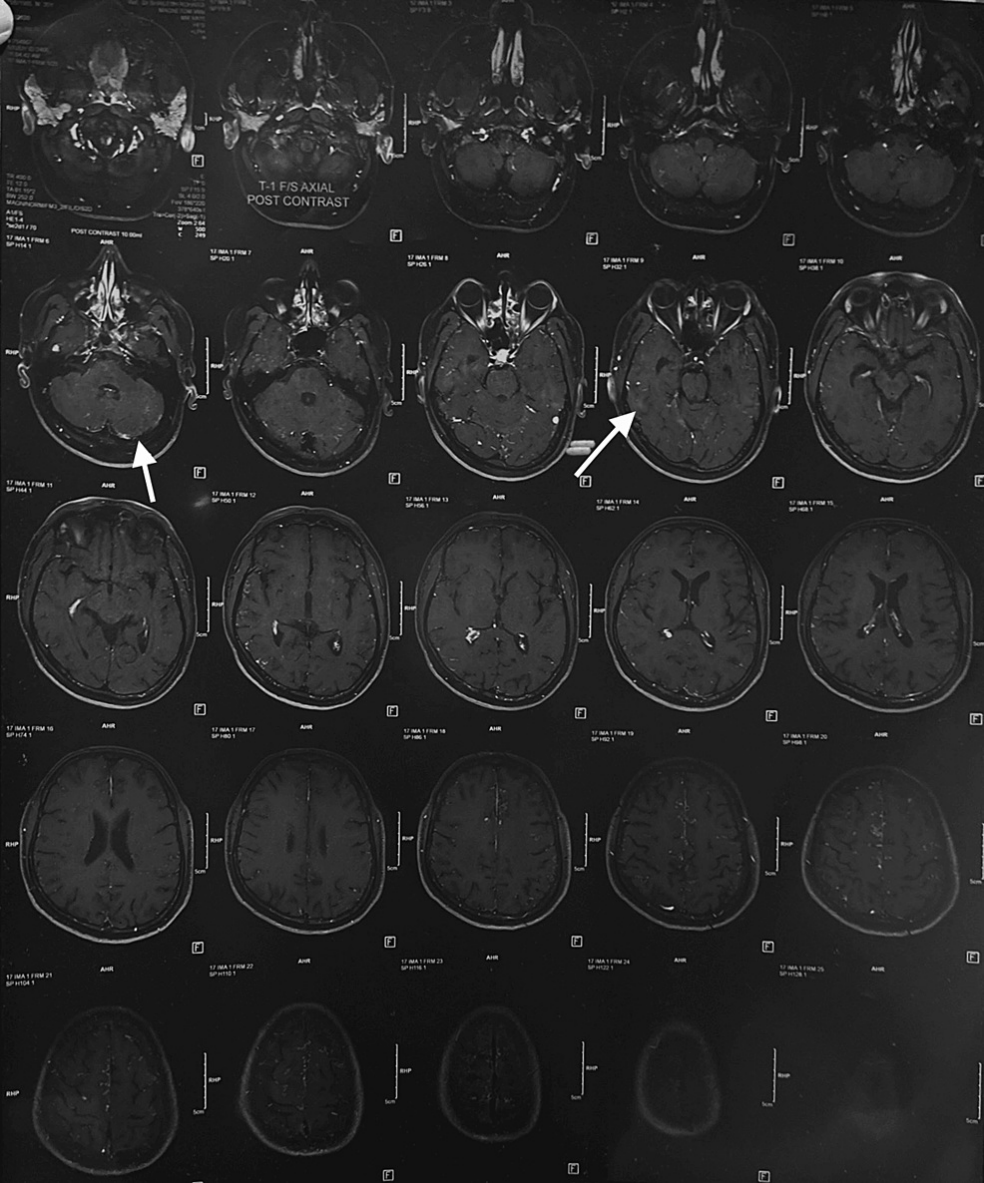
MRI brain (plain) demonstrating cortical white matter lesions with T2 hyperintensities

MRI brain was suggested and performed six months back and showed cortical white matter lesions with T2 hyperintensities (Figure 3).

**Figure 4:**
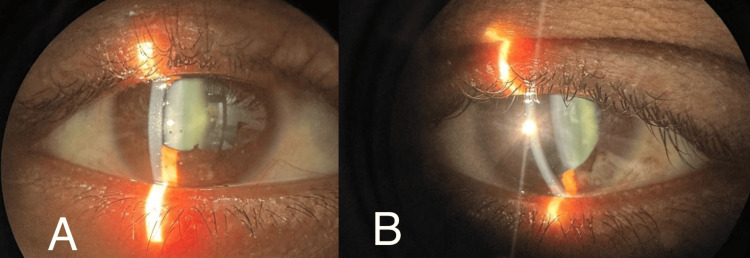
Image (A - right eye, B - left eye) showing slit lamp photograph depicting granulomatous keratic precipitates (KPs) in both eyes. The epithelial and stromal oedema subsided with the anterior chamber showing 1+ cells in the right eye and 0.5+ cells in the left eye. Symptoms and signs of uveitis including the mutton fat KPs subsided two to three weeks following the treatment

A diagnosis of bilateral ocular sarcoidosis was established. The patient was managed with topical eye suspension prednisolone acetate 1% four times a day and topical eyedrop timolol 0.5% twice a day for two weeks initially. The patient was reviewed regularly and the anterior uveitis was well controlled with the treatment in two weeks. One week later, he manifested with granulomatous KPs in both eyes but the epithelial and stromal oedema subsided with the anterior chamber showing 1+ cells in the right eye and 0.5+ cells in the left eye (Figure 4). Symptoms and signs of uveitis subsided two weeks following the treatment. The patient is on regular follow-up and has shown no relapse.

## Discussion

Ocular involvement in sarcoidosis has been identified and established since the early 1900s. Sarcoidosis is a chronic granulomatous multisystem disease, typically, mostly idiopathic. Bacterial, viral, fungal, and environmental factors, along with agents such as the COVID-19 vaccine, have been implicated in its aetiology. The differential diagnosis for conditions resembling sarcoidosis can include infectious diseases, autoimmune uveitis, and masquerade syndrome. Numerous studies have explored the diverse ocular manifestations of sarcoidosis [1].

Vaccine-induced uveitis exhibits variable pathogenesis, with mechanisms including a close analogy between vaccine peptides and self-peptides in the uvea, inflammatory damage caused by aluminium salts, direct viral load (in live and attenuated vaccines), and type 4 hypersensitivity. BNT162b2 is a modified RNA vaccine which encodes a membrane-anchored severe acute respiratory syndrome coronavirus 2 (SARS-CoV-2) protein, devoid of live virus. It has been suggested that the robust immune responses elicited by mRNA vaccines to battle the SARS-CoV-2 infection may lead to uveitis [2-4].

In a study executed by Tomkins-Netzer et al. in 2022, a larger proportion of patients had a previous history of uveitis (52%) and were recognised with anterior uveitis (90.96%) post-vaccination [6]. In cases reported to the Vaccine Adverse Event Reporting System, a small number of patients with VAU (vaccine-associated uveitis) had previously been detected with uveitis approximately 9.7% or systemic autoimmune diseases approximately 1.2%, and almost half of the patients (44.9%) were identified with anterior uveitis post-vaccination. Uveitis has also been reported following various vaccinations against hepatitis B virus, influenza virus, human papillomavirus (HPV), measles, mumps, rubella (MMR), typhoid, and others [4].

## Conclusions

In conclusion, we present an unconventional case of a 35-year-old male who exhibited an acute episode of anterior and posterior uveitis, which responded well to topical corticosteroids and beta blockers. The patient had a history of similar complaints in the past, as well as a COVID-19 vaccine administration two years prior. Additionally, he had been diagnosed with neurosarcoidosis two years ago and had experienced erythema nodosum one to two years ago, along with intermittent cough episodes, all of which had been thoroughly treated. Our case suggests that vaccine-induced uveitis is usually mild and typically responsive to topical corticosteroid treatment.

## References

[1] Pasadhika S, Rosenbaum JT (2015). Ocular sarcoidosis. Clin Chest Med.

[2] Numakura T, Murakami K, Tamada T (2022). A novel development of sarcoidosis following COVID-19 vaccination and a literature review. Intern Med.

[3] Cam F, Gok G, Celiker H (2023). Granulomatous anterior uveitis following mRNA-based COVID-19 vaccination: a case report. Indian J Ophthalmol.

[4] Singh RB, Parmar UP, Kahale F, Agarwal A, Tsui E (2023). Vaccine-associated uveitis after COVID-19 vaccination: Vaccine Adverse Event Reporting System database analysis. Ophthalmology.

[5] Hien DL, Onghanseng N, Ngoc TT (2020). Yet another case of ocular sarcoidosis. Am J Ophthalmol Case Rep.

[6] Tomkins-Netzer O, Sar S, Barnett-Griness O, Friedman B, Shyriaieva H, Saliba W (2022). Association between vaccination with the BNT162b2 mRNA coronavirus disease 2019 vaccine and noninfectious uveitis: a population-based study. Ophthalmology.

